# The Impact of 3D Prism Cavity for Enhanced Oil Recovery Using Different Nanomaterials

**DOI:** 10.3390/ma16114011

**Published:** 2023-05-27

**Authors:** Mudasar Zafar, Hamzah Sakidin, Iskandar Dzulkarnain, Abida Hussain, Mikhail Sheremet, Roslinda Nazar, Abdullah Al-Yaari, Nur Asyatulmaila Mohamad Asri, Shazia Bashir

**Affiliations:** 1 Department of Fundamental and Applied Sciences, Universiti Teknologi PETRONAS, Bandar Seri Iskandar 32610, Malaysia; hamzah.sakidin@utp.edu.my (H.S.); abida_20000262@utp.edu.my (A.H.); abdullah_20001447@utp.edu.my (A.A.-Y.); nur_17000559@utp.edu.my (N.A.M.A.); 2 Center for Research in Enhanced Oil Recovery, Universiti Teknologi PETRONAS, Bandar Seri Iskandar 32610, Malaysia; 3 Department of Petroleum Engineering, Universiti Teknologi PETRONAS, Bandar Seri Iskandar 32610, Malaysia; iskandar_dzulkarnain@utp.edu.my; 4 Laboratory on Convective Heat and Mass Transfer, Tomsk State University, 634050 Tomsk, Russia; michael-sher@yandex.ru; 5 Department of Mathematical Sciences, Faculty of Science and Technology, Universiti Kebangsaan Malaysia (UKM), Bangi 43600, Malaysia; rmn@ukm.edu.my; 6 Department of Physics and Applied Mathematics and Centre for Mathematical Sciences, Pakistan Institute of Engineering and Applied Sciences, Nilore 45650, Pakistan; shazia@pieas.edu.pk

**Keywords:** nanomaterials, enhanced oil recovery, reservoir geometry, 3D prism

## Abstract

Enhanced oil recovery (EOR) has been offered as an alternative to declining crude oil production. EOR using nanotechnology is one of the most innovative trends in the petroleum industry. In order to determine the maximum oil recovery, the effect of a 3D rectangular prism shape is numerically investigated in this study. Using ANSYS Fluent software(2022R1), we develop a two-phase mathematical model based on 3D geometry. This research examines the following parameters: flow rate Q = 0.01–0.05 mL/min, volume fractions = 0.01–0.04%, and the effect of nanomaterials on relative permeability. The result of the model is verified with published studies. In this study, the finite volume method is used to simulate the problem, and we run simulations at different flow rates while keeping other variables constant. The findings show that the nanomaterials have an important effect on water and oil permeability, increasing oil mobility and lowering IFT, which increases the recovery process. Additionally, it has been noted that a reduction in the flow rate improves oil recovery. Maximum oil recovery was attained at a 0.05 mL/min flow rate. Based on the findings, it is also demonstrated that Si$O_{2}$ provides better oil recovery compared to A$l_{2}O_{3}$. When the volume fraction concentration increases, oil recovery ultimately increases.

## 1. Introduction

In the energy sector, nanotechnology has demonstrated highly promising results in terms of retrieving the most oil from reservoirs. The addition of nanomaterials to the reservoir significantly increases the recovery rate, because this affects the wettability of the oil, reduces interfacial tension, and modifies fluid properties, which quickly mobilizes the oil from the reservoirs [1].

Primary, secondary, and improved oil recovery are the three stages of the oil recovery process. As the demand for energy rises and oil reservoirs are declining in number, a new challenge becomes apparent: how to fully utilize existing reservoirs to recover the maximum amount of oil. Unfortunately, only 35–65% of the oil is recovered in EOR, and there is a need to recover the remaining oil in order to meet the energy demand [2].

Nanoparticles (NPs) and nanofluids have a considerable impact in the domains of petroleum engineering and materials science as interdisciplinary sciences [3,4]. They have advanced significantly in recent decades, revealing their conceivable uses in EOR [5,6]. NPs and nanofluids have enhanced EOR processes in numerous studies over the last decade [7,8,9,10,11,12]. Various studies have used nanomaterials for mobility control, with outstanding results in reducing water snip, increasing sweep efficiency, and improving oil recovery [12,13,14,15,16,17]. Nanomaterials can change the water flow path in porous media by reducing capillary force and relative permeability. In addition, nanomaterials are resistant to destruction in high-salinity and high-temperature oil and gas reservoirs [18,19,20]. Surfactant solutions containing nanomaterials were also examined as nanofluids in several experiments to improve oil recovery in challenging reservoir environments [21].

Nanomaterials have been utilized by a few researchers in order to decrease the viscosity of bitumen as well as heavy and semi-heavy oil. According to the findings of various experiments, the NPs’ content, size, and type are three distinct characteristics that influence the process of lower viscosity in heavy oil [22,23,24,25]. Furthermore, extensive research has shown that the two primary mechanisms by which NPs have a high potential to increase oil recovery are wettability modification and IFT decrease between fluids and rocks [26,27,28,29,30].

Researchers [31,32] have found that adding titanium NPs to hydrocarbon-soaked sandstone made it possible to obtain 80% more oil out of the rock. Experts [33] have examined the efficiency of copper nanoparticles for oil recovery. The findings revealed that using copper nanoparticles boosted oil recovery by 71%. The authors of [34] studied how floods of carbon nanoparticles were altered. They discovered that carbon-based fluorescent nanoparticles (NPs) increase oil recovery in carbonate reservoirs by more than 96%.

The utilization of hydrophilic silica dioxide NPs for EOR at oil-, intermediate-, and water-wet reservoir temperatures was explored by researchers [35,36,37,38]. The findings of the experiments showed that NPs are stable at high temperatures and do not aggregate on porous surfaces. The authors of [39] investigated moist NPs (AEROSIL 200) with sodium dodecyl sulphate (SDS) surfactant in sandstone cores. The results demonstrated that injecting a NP-enriched surfactant (2500 ppm) into the core plug increases ultimate hydrocarbon recovery by 11%. The primary mechanisms underlying this oil recovery were the modification of wettability (from water-wet to oil-wet) by NPs and a diminution in IFT by surfactants and NPs. Injecting a NP-enriched surfactant into the core plug increased the eventual hydrocarbon recovery by 11%. The key processes driving this oil recovery included NPs changing the wettability (from water-wet to oil-wet) and the surfactant, as well as NPs decreasing IFT.

From the above literature, it is known that nanoparticles increase the oil recovery rate quite efficiently, but there has also been experimentally conducted research focused on mathematical modeling and reservoir geometry [40,41,42,43]. The literature also suggests that reservoir geometry plays a critical role in determining the effectiveness of EOR techniques [44,45]. The study of geometric effects can help determine the shape and size of the reservoir and the distribution of oil within it. This information is essential for developing and implementing effective EOR strategies [46]. Geometric effects can help identify the most effective EOR techniques for a given reservoir geometry. This includes techniques such as chemical flooding, thermal recovery, and gas injection. By understanding the impact of geometry on each technique, researchers can optimize the technique and maximize oil recovery. The geometric effect is essential for finding the maximum oil recovery rate in EOR. It helps to explain reservoir geometry, optimize EOR techniques, reduce uncertainty, and, ultimately, to maximize the efficiency and effectiveness of EOR.

In this paper, we develop and investigate a mathematical model for a 3D rectangular prism to determine the maximum oil recovery using two different nanomaterials, silicon dioxide and aluminum oxide. The effects of the flow rate, nanoparticle concentration, different porosity parameters, and relative permeability are also examined in a 3D rectangular prism. The reason for selecting a 3D rectangular prism is that this shape of geometry correlates with the real condition of the reservoir, and the findings are validated with published experimental work, which demonstrates the accuracy of this work.

## 2. Mathematical Model

A mathematical model for a 3D rectangular prism to predict maximum oil recovery is discussed in this section. In the flooding process, two different nanoparticles (i.e., SiO_2_ and Al_2_O_3_) are used.

### 2.1. Assumptions

The following assumptions are made in order to create the mathematical model:

i. There is only one-dimensional flow as it moves through the cavity.
ii. The rock in the reservoir is thought to be sandstone and tidy.
iii. The fluid that exits the cavity is incompressible.
iv. The Darcy Law is in effect during the flooding process.
v. The effect of chemical reactions is ignored.
vi. The flow inside the cavity is isothermal.
vii. The nanofluid flow is Newtonian, and the effect of gravity is ignored.

### 2.2. Geometry Creation

**Figure 1:**
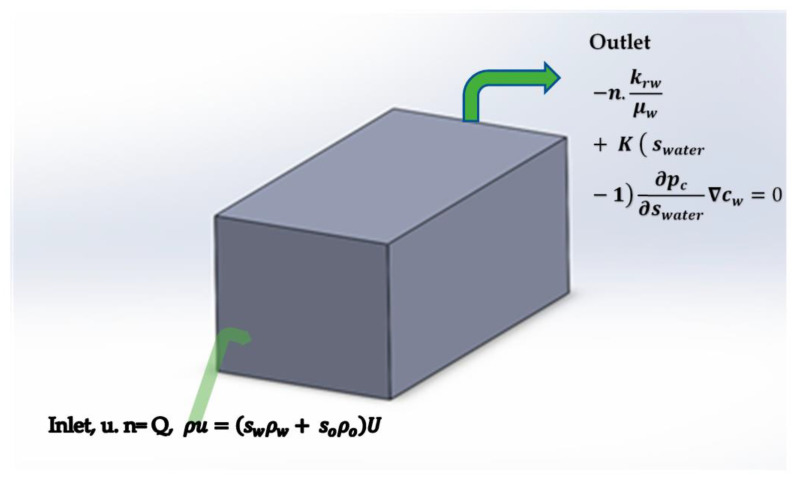
3D prism cavity used in flooding process.

It is very important to create accurate geometry for the reservoir simulation. In this study, a 3D rectangular prism cavity (see Figure 1) is used for the flooding process, and a simulation is performed in ANSYS Fluent (2022R1) software. The parameters and physical quantities that are used to formulate the geometry are provided in Table 1.

To extract oil inside the 3D rectangular prism, silica and aluminum nanoparticles are used in the nanoflooding method. Table 2 and Table 3 show the physical properties of these nanomaterials as well as the properties of the reservoir’s rock surface that was used in this simulation.

### 2.3. Mathematical Equations

The following system of nonlinear partial differential equations is combined to form a two-phase mathematical model for the 3D prism geometry [48,49].

The extended Darcy equation is as follows:

(1) ∇ρu=0,

where the values of velocity, density, and viscosity can be calculated as:

(2) $$u=\;\frac{k}{\mu }\;\frac{\partial p}{\partial x}$$

(3) $$\rho =s_{w\;}\rho_{w}+s_{o}\rho_{o}$$

(4) $$\frac{1}{\mu }=s_{water\;}\frac{k_{rw}}{\mu_{rw}}+s_{oil\;}\frac{k_{r0}}{\mu_{o}}$$

The saturation equation is as follows:

(5) $$\frac{\partial }{\partial x}\left(c_{w}u\right)=\frac{\partial }{\partial x}[\left(D_{c}\frac{\partial }{\partial x}c_{w}\right)]$$

(6) $$c_{w}=s_{water\;}\rho_{water}$$

(7) $$D_{c}=\frac{k_{rw}}{\mu_{w}}+K\;\left(s_{water}-1\right)\frac{\partial p_{c}}{\partial s_{water}}$$

We compute the velocity, pressure, and saturation of oil and water using Equations (1)–(7). In the above model, φ defines porosity, ρ is for density, *u* represents fluid initial velocity, *k* is for thermal conductivity, μ defines viscosity, p stands for pressure, t  is for time, K denotes relative permeability, and $D_{c}$ defines the diffusion coefficient. In addition to this subscript, *w* is for water and *o* is for oil, with rw giving residual water and r0 residual oil.

The Brooks–Corey [50] and logarithmic models are used to determine the capillary pressure.

(8) $$\rho_{c}=-B_{C}\times \log\left(S_{e}\right)$$

where $B_{C}$ is the effective capillary pressure parameter, and $S_{e}$ can be calculated using Equation (9):

(9) $$S_{e}=\frac{s_{w}-s_{wr}}{1-s_{or}-s_{wr}}$$

In Equation (9), $S_{e}$ is said to be effective water saturation.

For the nanoparticle concentration equation:

The mass transport of the nanofluid is defined by Equation (10) [19,49]:

(10) $$\frac{\partial \varphi S_{w}\psi_{w}}{\partial t}+u_{w}\cdot \frac{\partial \psi_{w}}{\partial x}=\frac{\partial }{\partial x}\cdot \left(\varphi S_{w}\psi_{w}D_{w}\frac{\partial \psi_{w}}{\partial x}\right)-R_{\iota }$$

The values of $R_{\iota }$ can be calculated using Equation (8).

(11) $$R_{\iota }\;=\frac{\partial \omega }{\partial t}+\frac{\partial \omega *}{\partial t}$$

The terms ω  and ω* be calculated using the following [51,52] relation:

(12) $$\frac{\partial \omega }{\partial t}=K_{d}\upsilon C,\;\frac{\partial \omega *}{\partial t}=K_{p}\upsilon C$$

For the porosity and relative permeability equations:

The porosity can be calculated by the equation defined below:

(13) $$\phi =\phi_{initail}-\sum {𝓸}_{i}^{*}+{𝓸}_{i}$$

In the two-phase nanoflooding process for EOR, the relative permeability can be determined using the given relation introduced by [53].

(14) $$K_{rw,P}=\left(\;1-\Psi_{s}\right)K_{rw}+X_{S}K_{rw,\;C}$$

(15) $$K_{ro,P}=\left(\;1-\Psi_{s}\right)K_{ro}+X_{S}K_{ro,\;C}$$

where the term $X_{S}$ can be calculated as:

(16) $$X_{S}=\frac{S_{RPt}}{S_{SC}}$$

The values of $S_{RPt}$ and $S_{SC}$ are determined by:

(17) $$S_{RPt}=\beta \sum {𝓸}_{i}^{*}+{𝓸}_{i}\frac{6}{d_{p}}$$

(18) $$S_{SC}=7000\phi \sqrt{\frac{\phi }{K}}$$

The relative permeability is measured in ratio, which does not have a SI unit, and its values are between 0 and 1.

### 2.4. Initial and Boundary Conditions

The initial and boundary conditions that are used in this problem are given below and defined in Table 4 in detail.

(19) $$\mathrm{When}\;t=0,\;\;\mathrm{the}\;\mathrm{initial}\;\mathrm{saturation}\;\mathrm{of}\;\mathrm{water}\;\mathrm{is}\;\mathrm{zero},\;\mathrm{i.e.},\;s_{w}^{0}=0.$$

(20) −n·ρu=0

(21) −n·q=0

(22) $$\rho u=\left(s_{w}\rho_{w}+s_{o}\rho_{o}\right)U$$

(23) $$-n\cdot D_{C}\nabla c_{w}=0$$

(24) $$\mathrm{At}\;t=0,\;s_{w}=0.10$$

(25) $$\mathrm{At}\;t=0,\;\left\{\begin{array}{c} \Psi =0\\ \;\Psi =\Psi_{i} \end{array}\right.$$

(26) $$\mathrm{At}\;t=0,\;\left\{\begin{array}{c} \omega =0\\ \;\omega_{i}=0 \end{array}\right.$$

### 2.5. Mesh Test

An analysis of the mesh to choose the optimal mesh size to perform the simulation is very important. In this study, we performed several experiments to select the best mesh. The sizes of different meshes during the simulation are provided in Table 5.

**Figure 2:**
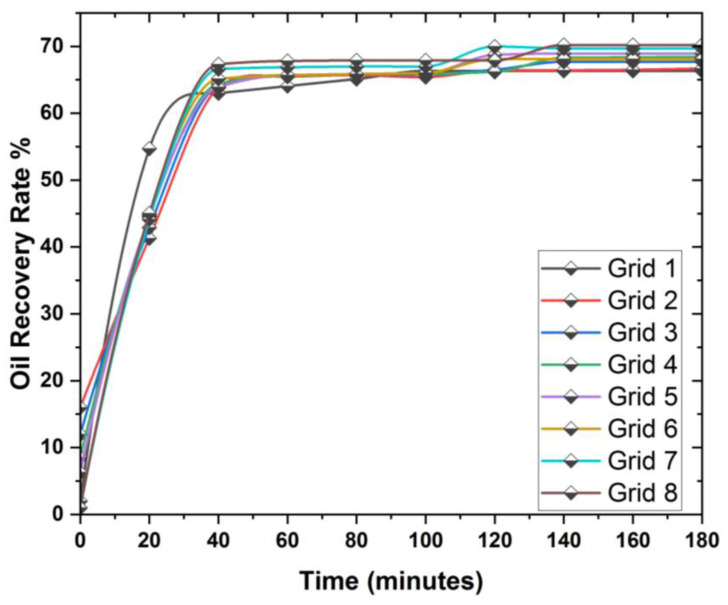
Graphical comparison of mesh analysis in 3D prism.

**Figure 3:**
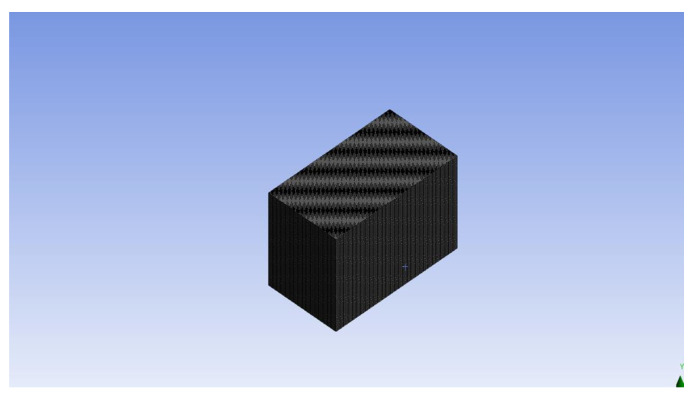
Mesh used in current study to find oil recovery rate.

The effects of the grid’s dependence on geometry are seen in Figure 2. As observed, grids 6, 7, and 8 are like one another. This indicates that the model is unaffected by the mesh size. The optimum mesh to use is grid number 8, which has 325,230 nodes. The mesh used in this problem is presented in Figure 3.

After choosing the mesh, we must now compare the model results with previous experimental findings. This is done in the next subsection.

### 2.6. Experimental Validation

The model’s reliability can be determined by comparing its predictions to the results of a previously performed experiment [54]. In this paper, Si$O_{2}$ and Al_2_O_3_ were added to a porous rectangular prism to enhance oil outflow. Table 6 displays the experimental conditions and rock core characteristics.

**Figure 4:**
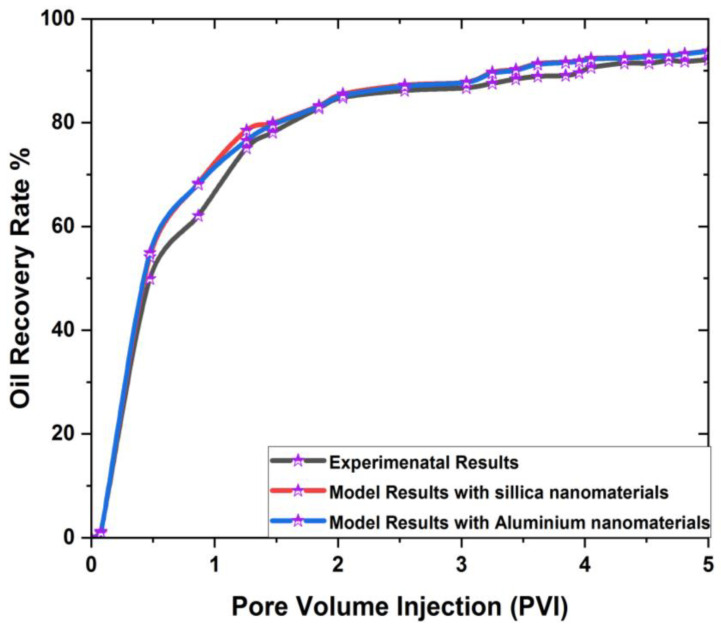
Validation of computational results with experimental results [54].

Figure 4 demonstrates that the simulation’s result complies with the experimental data for estimating oil recovery. We will explain the results in the next section.

## 3. Results

In this research, we use ANSYS Fluent’s finite volume approach to model the influence of a 3D rectangular prism on a reservoir filled with Si$O_{2}$ and Al_2_O_3_ nanomaterials to determine the greatest percentage of oil recovery possible. The effects of the nanomaterials on relative permeability change and oil recovery are studied, along with the effects of flow rate Q = 0.01 to 0.05 mL/min, porosities Φ = 0.1 to 0.4, and nanoparticle volume fraction Ψ = 0.01 to 0.05%.

**Figure 5:**
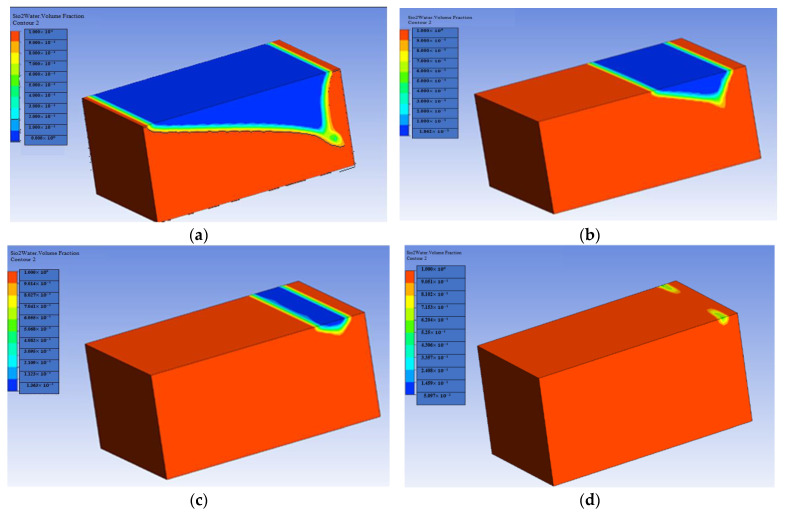
Flow rate effect on oil recovery in the presence of Si$O_{2}$: (**a**) Q = 0.01 mL/min; (**b**) Q = 0.02 mL/min; (**c**) Q = 0.03 mL/min; (**d**) Q = 0.05 mL/min.

**Figure 6:**
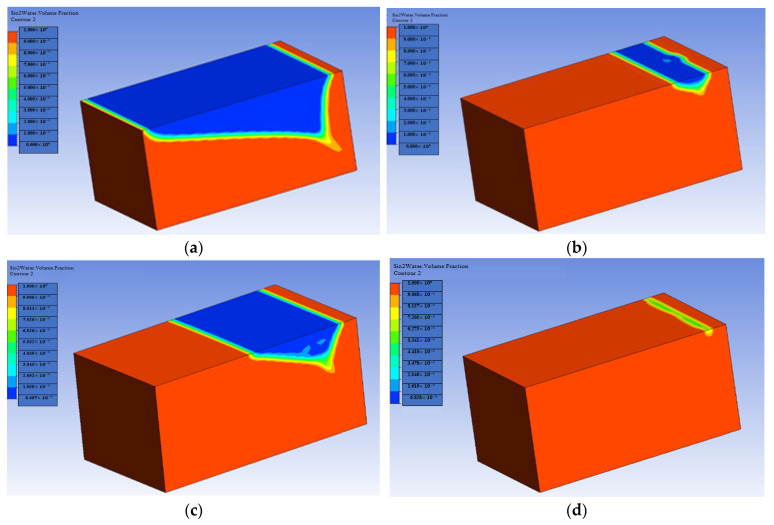
Flow rate effect on oil recovery in the presence of Al_2_O_3_: (**a**) Q = 0.01 mL/min; (**b**) Q = 0.02 mL/min; (**c**) Q = 0.03 mL/min; (**d**) Q = 0.05 mL/min.

**Figure 7:**
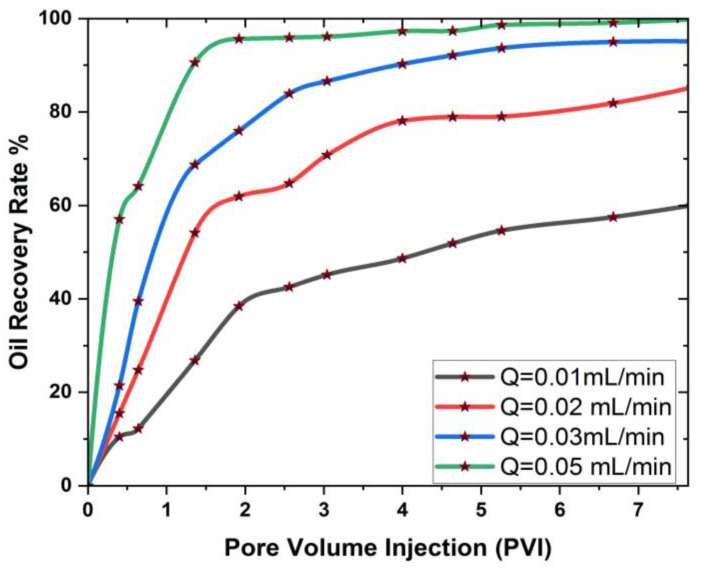
Graphical analysis of the effect of flow rate on oil recovery with Si$O_{2}$.

**Figure 8:**
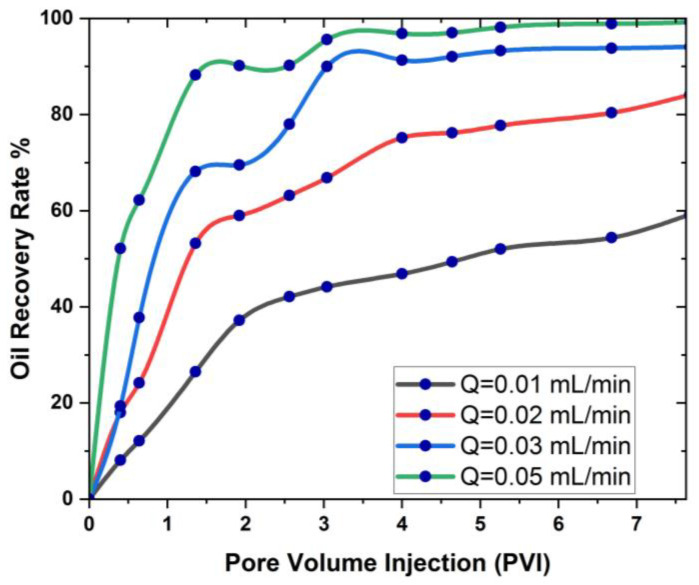
Graphical analysis of the effect of flow rate on oil recovery with Al_2_O_3_.

Figure 5 and Figure 6 show the cantor analysis of oil recovery at different pore volumes at different flow rates due to Si$O_{2}$ and Al_2_O_3_ nanoparticles. It is observed that as the flow rate decreases, the amount of oil recovery increases, because, at Q = 0.05 mL/min, the maximum oil is recovered due to an increase in the time period; as the simulation time increases, the oil rate increases. When the flow rate decreases, the injected fluid can move around the reservoir more quickly, resulting in a greater displacement of trapped oil, which contributes to increased oil recovery. This is due to the fact that the flow rate is reduced, allowing for increased hydrocarbon recovery. In particular, as flow rates increase as a result of this, the injected fluid leaps over the reservoir rock, resulting in a decrease in oil recovery. Another reason is due the fact that when the flow rate decreases, there is an increase in contact time that results in the maximum interaction of the molecules, and hence an increase in oil recovery. A similar observation is reported by the authors of [55,56]. A graphical comparison of the flow rate on oil recovery in the presence of Si$O_{2}$ and Al_2_O_3_ is also provided in Figure 7 and Figure 8.

Figure 7 and Figure 8 show that the flow rate has a favorable effect on oil recovery at each pore volume, with a steady increase in oil recovery showing the flow rate’s optimal influence on the 3D cavity. This means that the flow rate must be extremely low in order to increase the fluid flow and, as a result, the rate of oil recovery. It is also obvious from Figure 7 and Figure 8 that oil recovery is greater in the presence of Si$O_{2}$ than Al_2_O_3_.

**Figure 9:**
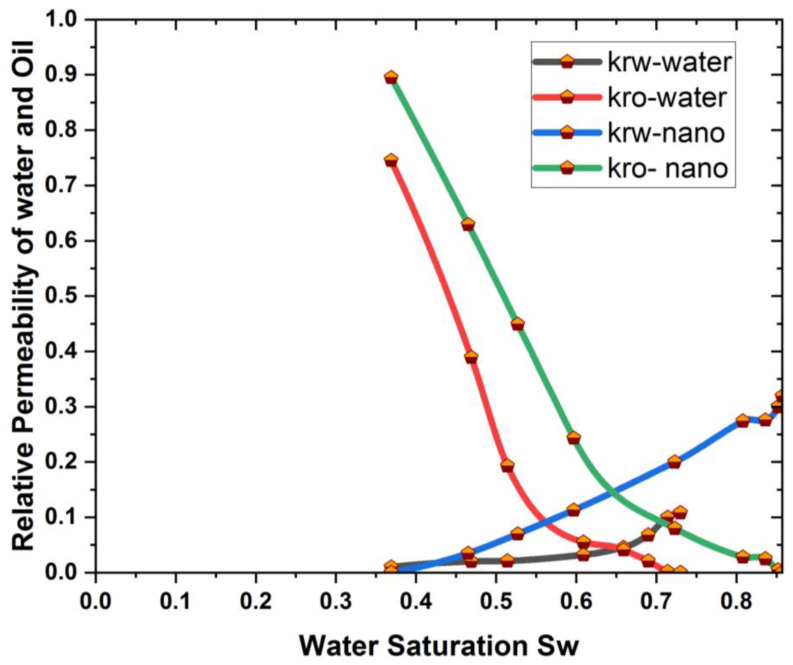
Influence of nanomaterials on relative permeability [57].

The impact of the nanomaterials on relative permeability is shown in Figure 9. It is observed that the nanomaterials change the permeability, which increases the oil recovery rate in comparison to the permeability due to water flooding and nanoflooding [57].

The effect of the relative permeability on oil recovery in the geometry of the rectangular prism is investigated, and it is observed that the nanofluids have the ability to enhance the oil recovery process, because the ability of a fluid to pass through a porous medium (such as a reservoir rock) relative to another fluid is referred to as relative permeability. There may be numerous fluids present in an oil reservoir, including oil, water, and gas. The relative permeability of each fluid is governed by the reservoir rock features, fluid properties, and fluid flow rate. Nanoparticles were put into the reservoir in this study to modify the relative permeability of the oil and water phases. The nanoparticles reduce the friction between the oil and water phases. This decreases the capillary forces that retain oil in reservoirs, making recovery easier. Similar results were also reported by the authors of [58,59,60,61].

**Figure 10:**
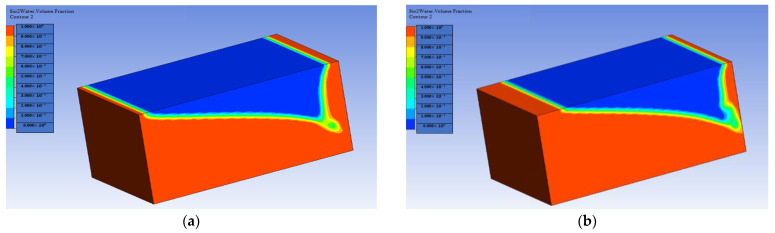

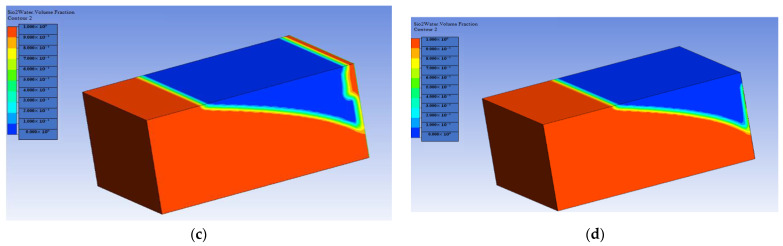
Volume fraction effect on oil recovery in the presence of Si$O_{2}$: (**a**) Ψ=0.01%; (**b**) Ψ=0.02%; (**c**) Ψ=0.03%; (**d**) Ψ=0.04%.

**Figure 11:**
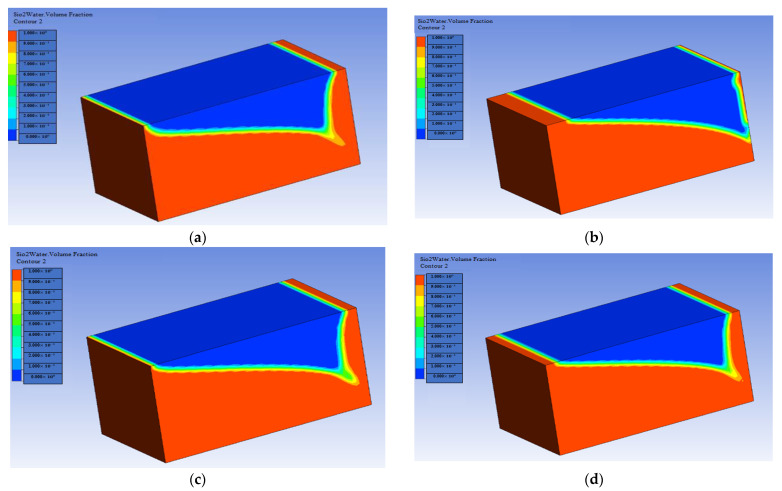
Volume fraction effect on oil recovery in the presence of Al_2_O_3_: (**a**) Ψ=0.01%; (**b**) Ψ=0.02%; (**c**) Ψ=0.03%; (**d**) Ψ=0.04%.

Figure 10 and Figure 11 show that concentrations of the nanoparticles have a favorable effect on oil recovery at each pore volume, with a steady increase in oil recovery showing that the volume fraction has an optimal influence on the 3D cavity.

**Figure 12:**
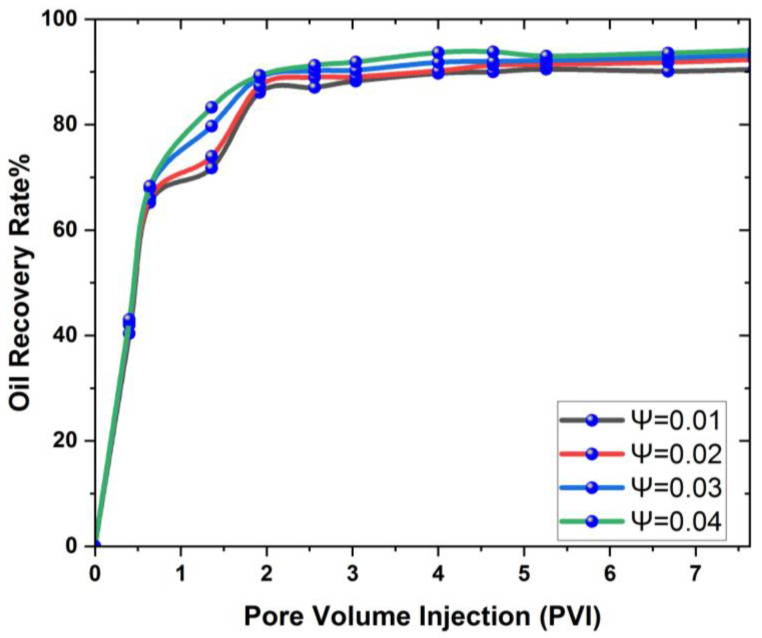
Graphical comparison of volume fraction effect on oil recovery with Si$O_{2}$.

**Figure 13:**
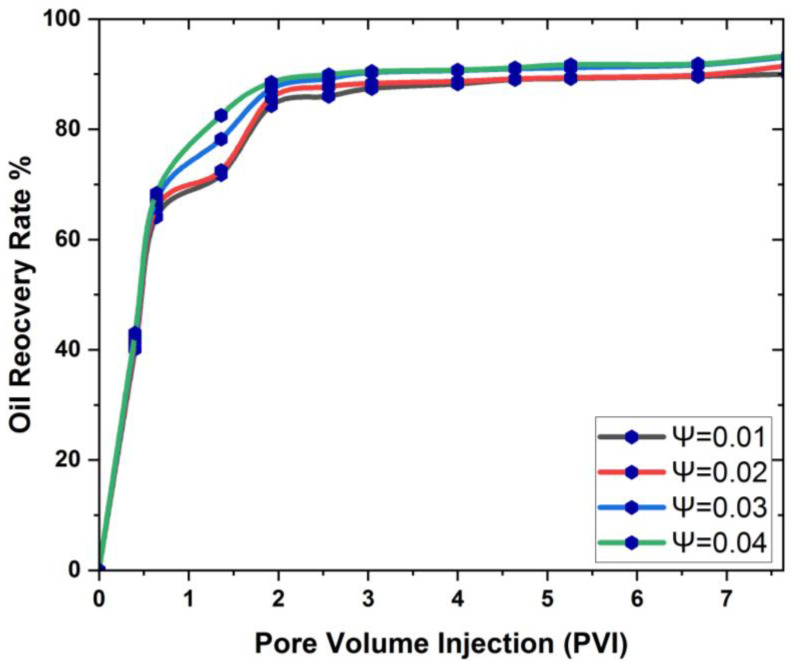
Graphical comparison of volume fraction effect on oil recovery with Al_2_O_3_.

Figure 8 and Figure 9 clearly show that as the concentration of nanoparticles increases, so does the rate of oil recovery, and the maximum feasible oil recovery is attained in both nanoparticles. This is because an increase in concentration reduces the interfacial tension (IFT) between the oil and the injected fluid, allowing the oil to be mobilized and the recovery rate to increase dramatically. Researchers have reported the same phenomenon of increased oil recovery due to an increase in nanoparticle concentration [62,63]. In addition to this, a graphic comparison of the improved oil recovery due to Si$O_{2}$ and Al_2_O_3_ is also provided in Figure 12 and Figure 13.

Based on the results mentioned above, it is indicated that when the concentration of nanoparticles in the 3D prism cavity grows, the rate of oil recovery increases, and the highest oil recovery reached is 99.24% at Ψ = 0.04.

## 4. Discussion

The rate of oil production in the world’s largest oil sources is decreasing, whereas daily demand for oil has risen to a million barrels. The decreasing supply of oil reserves has compelled the petroleum industry to investigate new oil reserves and develop innovative techniques for producing more oil from existing petroleum reservoirs. This study employs Si$O_{2}$ and Al_2_O_3_ nanomaterials to create a mathematical model for a three-dimensional, rectangular, porous cavity. According to the literature, reservoir geometry plays a crucial role in oil recovery; however, limited numerical research has been conducted to explain the phenomenon of oil recovery by numerically analyzing the influence of reservoir geometry. The primary objective of this article was to determine the oil recovery rate in 3D rectangular prism geometry, and the results indicate that the effect of this 3D geometry increases oil recovery. The computational results were obtained using a finite volume ANSYS solver and compared with the experimental results obtained by [54]. It was found that the existing results yield a better oil recovery rate. The use of FVM has the advantage of discretizing the reservoir into small control volumes. This method gives a realistic description of the reservoir’s shape and physical properties, as well as the ability to easily incorporate boundary conditions such as injection rates and pressures, which improves the model’s ability to estimate the oil recovery rate.

The findings suggest that as the cavity flow rate decreases, Si$O_{2}$ and Al_2_O_3_ improve oil recovery in rectangular prism cavities. When the mass flow rate is 0.05 mL/min or 0.03 mL/min, the cavity extracts the most oil, as opposed to 0.01 mL/min and 0.02 mL/min. In the presence of a flow rate, the 3D rectangular prism cavity recovers 6.3% more oil than the Al_2_O_3_, demonstrating its worth.

This study additionally points out that nanofluids change the relative permeability of the water and oil phases in both cavities, increasing the rate of oil recovery. When Si$O_{2}$ and Al_2_O_3_ are injected, the maximum oil recovery for a 0.04% volume fraction of nanoparticles is 99.39% and 98.01%, respectively, in a 3D rectangular prism. Based on these findings, it is advised that, in the future, this research be expanded to include the use of ionic liquid nanoparticles and hybrid nanoparticles to maximize oil recovery.

## 5. Conclusions

In this article, a 3D rectangular prism cavity was used for a reservoir simulation to find the oil recovery rate using two different nanomaterials. The geometric effects can help to identify the most effective EOR techniques for a given reservoir geometry. By understanding the impact of geometry on each technique, researchers can optimize the technique and maximize oil recovery. The geometric effect is essential for finding the maximum oil recovery in EOR. The effect of flow rates and nanomaterials on relative permeability and different parameters of the concentrations of nanomaterials were investigated in the presence of silicon and aluminum nanoparticles, which give maximum oil recovery. Based on the findings, the following conclusions can be drawn:

- The nanoparticles introduced the relative permeability of the oil and water phases into the cavity change, reducing friction between the two. This reduces the capillary forces that keep oil in reservoirs and makes recovery easier.
- The oil recovery rate increases as the flow rate decreases, and the maximum amount of oil recovered at Q = 0.05 mL/min is 99.1% in the case of Si$O_{2}$, which shows that the effect of flow is very important in reservoir geometry to obtain maximum oil recovery.
- It is also observed that with an increase in the nanoparticle concentration at each pore volume injection, the oil recovery rate also increases.
- It was also discovered that the reservoir’s shape has a substantial impact on oil recovery enhancement, since it directly influences flow behavior, which can increase oil recovery.
- The findings also indicate that Si$O_{2}$ provides a 6.3% higher recovery than Al_2_O_3_.

## Figures and Tables

**Table 1:** Parameters to construct the 3D prism.

	Physical Quantities	Quantities
Parameter of the geometry	Largest width	0.30 m
	In radius	0.12 m
	Circumference	0.15 m
	Smallest width	0.25 m
	Length of the side	0.14 m
Volume and cross-sectional area	Core volume	0.49 $m^{3}$
	Inlet cross sectional area	0.45 $m^{2}$
Physical properties	Inlet temperature of the fluid	300 K
	Initial temperature in cavity	290 K
	Initial input pressure	1 atm
	Final output pressure	1 atm

**Table 2:** Properties of nanoparticles studied in 3D prism [18,19].

Nanomaterials/Properties	Physical Property	Value
Si$O_{2}$	Density	2220 $\mathrm{kg}/m^{3}$
	Heat capacity	745${\;\mathrm{JKg}}^{-1}K^{-1}$
	Thermal conductivity	36 ${\mathrm{Wm}}^{-1}K^{-1}$
	Volume fraction	0.01
	Diameter	40 nm
	Molecular mass	60 nm
Al_2_O_3_	Density	3970 $\mathrm{kg}/m^{3}$
	Heat capacity	765 ${\mathrm{JKg}}^{-1}K^{-1}$
	Thermal conductivity	36 ${\mathrm{Wm}}^{-1}K^{-1}$
	Volume fraction	0.01
	Diameter	40 nm
	Molecular mass	101.96 nm
Properties of Oil	Density	829 $\mathrm{kg}/m^{3}$
	Heat capacity	1670$\;{\mathrm{JKg}}^{-1}K^{-1}$
	Thermal conductivity	0.13 ${\mathrm{Wm}}^{-1}K^{-1}$
	Viscosity	4.5 × 10^−4^ Pa·s
Properties of Water	Density	990 $\mathrm{kg}/m^{3}$
	Heat capacity	4200$\;{\mathrm{JKg}}^{-1}K^{-1}$
	Thermal conductivity	0.6 ${\mathrm{Wm}}^{-1}K^{-1}$
	Viscosity	$10^{-3}$ Pa·s

**Table 3:** Properties of reservoir [47].

	Physical Properties	Values
Reservoir rock	Rock density	2714 kg$/m^{-3}$
Mesh size	Diameter	3 μm

**Table 4:** Boundary conditions of the problem.

Boundary Points	Boundary Conditions	Flow Boundary
Boundary 1	u · n = Q $\rho u=\left(s_{w}\rho_{w}+s_{o}\rho_{o}\right)U$ $\left\{\begin{array}{c} \Psi =0\\ \;\Psi =\Psi_{i} \end{array}\right.$	Inlet
Boundary 2	−n·ρu=0	No flow enters or leaves
Boundary 3	−n·ρu=0	No flow enters or leaves
Boundary 4	$-n\cdot D_{C}\nabla c_{w}=0$	Outlet

**Table 5:** Grid analysis for 3D prism.

Grid Number	1	2	3	4	5	6	7
Grid Size	30	3000	2456	4802	13,403	30,251	325,230

**Table 6:** Characteristics of the core plugs employed by [54].

Properties	Range with SI
Diameter	4.15 cm
Length	5.78 cm
Permeability	110.4 mD
Porosity	17.5%

## Data Availability

Not applicable.
